# Induction of Apoptosis and Antiproliferative Activity of Naringenin in Human Epidermoid Carcinoma Cell through ROS Generation and Cell Cycle Arrest

**DOI:** 10.1371/journal.pone.0110003

**Published:** 2014-10-16

**Authors:** Md Sultan Ahamad, Sahabjada Siddiqui, Asif Jafri, Sheeba Ahmad, Mohammad Afzal, Md Arshad

**Affiliations:** 1 Department of Zoology, Shibli National (PG) College, Azamgarh, Uttar Pradesh, India; 2 Molecular Endocrinology Lab, Department of Zoology, University of Lucknow, Lucknow, Uttar Pradesh, India; 3 Department of Zoology, D S College, Aligarh, Uttar Pradesh, India; 4 Human Genetics and Toxicology Laboratory, Department of Zoology, Aligarh Muslim University, Aligarh, Uttar Pradesh, India; Aligarh Muslim University, India

## Abstract

A natural predominant flavanone naringenin, especially abundant in citrus fruits, has a wide range of pharmacological activities. The search for antiproliferative agents that reduce skin carcinoma is a task of great importance. The objective of this study was to analyze the anti-proliferative and apoptotic mechanism of naringenin using MTT assay, DNA fragmentation, nuclear condensation, change in mitochondrial membrane potential, cell cycle kinetics and caspase-3 as biomarkers and to investigate the ability to induce reactive oxygen species (ROS) initiating apoptotic cascade in human epidermoid carcinoma A431 cells. Results showed that naringenin exposure significantly reduced the cell viability of A431 cells (p<0.01) with a concomitant increase in nuclear condensation and DNA fragmentation in a dose dependent manner. The intracellular ROS generation assay showed statistically significant (p<0.001) dose-related increment in ROS production for naringenin. It also caused naringenin-mediated epidermoid carcinoma apoptosis by inducing mitochondrial depolarization. Cell cycle study showed that naringenin induced cell cycle arrest in G_0_/G_1_ phase of cell cycle and caspase-3 analysis revealed a dose dependent increment in caspase-3 activity which led to cell apoptosis. This study confirms the efficacy of naringenin that lead to cell death in epidermoid carcinoma cells *via* inducing ROS generation, mitochondrial depolarization, nuclear condensation, DNA fragmentation, cell cycle arrest in G_0_/G_1_ phase and caspase-3 activation.

## Introduction

Apoptosis plays a crucial role in the normal development and pathology of a wide variety of tissues [1]. However, most cancer cells do not undergo apoptosis due to impairment of apoptotic signal transmission [2]. Triggering apoptosis in tumor cells can therefore be an effective strategy in anticancer therapy. Apoptosis can be initiated through two different mechanisms, the extrinsic death-receptor dependent pathway and a mitochondria-dependent or intrinsic pathway [3]. The extrinsic pathway is activated by cell-surface ligand-gated death receptors such as the tumor necrosis factor receptor super-family. Intrinsic apoptosis is initiated by disparate extracellular and intracellular stimuli. Mitochondrial release of cytochrome *c* directly triggers caspase-3 activation. Caspase-3 then cleaves cellular targets to promote apoptosis [4], [5].

Over 4000 different flavonoids have been identified in the human diet and the daily intake ranges from 23 mg to 1 g [6]. It shows an inverse association between consumption of flavonoids and the risk of human cancers at many sites [7]. The flavanone naringenin, especially abundant in citrus fruits such as grapefruit (*Citrus paradisi*), oranges (*Citrus sinensis*) and in tomato (*Solanum lycopersicum*) [8], is reported to have anti-proliferative effects in different cancer cell lines [9]. Generally cytotoxic and antitumor agents are non-selective and also kill normal proliferating cells. Identification of active cancer specific compounds remains a thrust area in drug screening and drug discovery mechanisms. Naringenin has been reported to induce apoptosis in various human cancer cells [10], [11] and its higher concentrations (>300 µM) cause normal cell toxicity in human lung embryonic fibroblasts. [12].

Oxidative stress induced DNA damage or deficient DNA-repair is reported to have an etiological or prognostic role in cancer [13]. Research also suggests that electrophiles could play a key role in chemical carcinogenesis. The oxidative inactivation of enzymes by free radicals and the accumulation of oxidized proteins may play a critical role in the alteration of cellular function and cell death. Oxidative damage can generate large amounts of carbonyl products and hence measurement of these components could reflect oxidative protein damage [14]. However, the mechanism of action of naringenin to suppress cell growth is still ambiguous, since this compound appears to have multiple cellular targets including cytochrome P450 enzymes [15].

Several mechanisms have been proposed for naringenin induced anti-proliferative effects i.e. antioxidant activities, kinase and glucose uptake inhibition, and to hamper cell proliferation *via* estrogen receptor [16], [17]. However, the composite (combined) involvement of reactive oxygen species (ROS), cell apoptosis, DNA fragmentation, mitochondrial membrane potential (MMP), cell cycle kinetics and caspase-3 activities in the molecular mechanisms of naringenin-induced anti-proliferative effects in human epidermoid carcinoma A431 cell line remains to be investigated. The aim of the present study was two-fold: (a) to analyze the anti-proliferative and apoptotic effects of naringenin through ROS mediated 3-(4,5-dimethylthiazol-2-yl)-2,5-diphenyltetrazolium bromide (MTT), nuclear condensation, mitochondrial membrane potential and DNA fragmentation, and (b) cell cycle kinetics and caspase-3 induction as biomarkers in cancer cell *viz.* human epidermoid carcinoma A431 cell.

## Materials and Methods

### Cell line culture

Normal skin cell (HaCaT) and Human epidermoid carcinoma (A431) cell line were obtained from Cell Repository, National Centre for Cell Sciences (NCCS), Pune, India. A431 was cultured in Dulbecco's modified eagle medium (DMEM) and HaCaT cell was cultured in DMEM:F12 (1∶1) medium supplemented with 10% (v/v) fetal calf serum (Himedia), 2.0 mM L-glutamine,1.5 g/l NaHCO_3_, 0.1 mM non-essential amino acids, 1.0 mM sodium pyruvate and 1% antibiotic solution. Cells were maintained at 37°C, 5% CO_2_ in a humidified air.

### MTT assay for cell viability in HaCaT and A431 cells

This assay is based on the enzymatic reduction phenomenon of 3-(4,5-dimethylthiazol-2-yl)-2,5-diphenyltetrazolium bromide (MTT) dye and provides a direct relationship between the viable cells and absorbance. The effect of naringenin on cell viability was evaluated by MTT reduction assay as performed earlier [18]. Selective doses *viz.* 50 µM, 100 µM, 200 µM, 300 µM, 400 µM, 500 µM and 750 µM of naringenin were prepared in dimethyl sulfoxide (DMSO) in final volume of 100 µl media. After 21 h exposure, 10 µl of MTT solution (5 mg/ml stock solution) was added in each well re-incubated for 3 h at 37°C until formazan blue crystal developed. Media was discarded from each well and 100 µl of DMSO was added to dissolve formazan crystals for 10 min at 37°C. The absorbance was recorded at 540 nm by microplate reader (BIORAD-680) and relative percentage cell viability was evaluated.

### Cell morphology analysis

The effect of naringenin was analyzed for morphological changes in the cultured cells [19]. The cells were seeded at a density of 1×10^4^ cells/well in 96-well culture plate. After overnight incubation, the cells were treated with different concentrations of naringenin for 24 h. The cellular morphology was observed under inverted phase contrast microscope (Nikon ECLIPSE Ti-S, Japan).

### Reactive oxygen species (ROS) activity assay

Microscopic fluorescence imaging was used to study ROS generation in A431cells after exposure to different concentrations of naringenin [20]. Cells (1×10^4^ per well) were seeded as described above for the MTT assay. Cells were then exposed to 50 µM, 100 µM, 200 µM, 300 µM, 400 µM, 500 µM and 750 µM concentrations of naringenin for 12 h. Cells were incubated with 2,7-Dichlorodihydrofluorescein diacetate (DCFH-DA) (10 mM) for 30 min at 37°C. The reaction mixture was aspirated and replaced by 200 µl of phosphate-buffered saline (PBS) in each well. The plate was kept on a shaker for 10 min at room temperature in the dark. An inverted fluorescent microscope (Nikon ECLIPSE Ti-S, Japan) was used to visualize intracellular fluorescence of cells and to capture images. For quantitative ROS analysis, cells (1×10^4^ per well) were re-seeded in 96-well black bottom culture plate and allowed to adhere for 24 h in a CO_2_ incubator at 37°C. A431 cells were treated with different concentrations of naringenin for 12 h. After exposure, cells were incubated with DCFH-DA (10 mM) for 30 min at 37°C. Fluorescence intensity was measured by multiwell micro-plate reader (Synergy H1 Hybrid Multi-Mode Microplate Reader, BioTek) at excitation wavelength of 485 nm and emission wavelength of 528 nm. Values were expressed as the percentage of fluorescence intensity relative to the control wells.

### DAPI staining for apoptosis analysis

The apoptotic effect of compounds was analyzed by using florescent nuclear dye 4′,6-diamidino-2-phenylindole dihydrochloride (DAPI) [21]. The cells were seeded and treated as mentioned previous. Cells were then washed with PBS and fixed in 4% paraformaldehyde for 10 min. Subsequently the cells were permealized with permealizing buffer (3% paraformaldehyde and 0.5% Triton X-100) and stained with DAPI dye. After staining, the images were captured and numbers of cells were quantified using a fluorescent microscope (Nikon ECLIPSE Ti-S, Japan).

### Assessment of mitochondrial membrane potential

Flouroprobe 5,5′,6,6′-tetrachloro-1,1′,3,3′-tetraethylbenzimidazol-carbocyanine iodide (JC-1) is a cationic, lipophilic dye and therefore has been extensively used to study the loss of the mitochondrial membrane potential which occurs during apoptosis [22]. In normal cells, due to high membrane potential (polarized mitochondria), the dye concentrates in the mitochondrial matrix, and it forms red fluorescent aggregates (J-aggregates). Any event that dissipates the mitochondrial membrane potential (depolarized mitochondria) prevents the accumulation of the JC-1 dye in the mitochondria and thus, the dye is dispersed throughout the entire cell leading to a shift from red (J-aggregates) to green fluorescence (JC-1 monomers). A decrease in red/green ratio is indicative of apoptosis. The cells were grown in 24-well plate and treated with different concentrations of naringenin. After 24 h exposure, the treated cells were washed with PBS and stained with 2 µg/ml of JC-1 dye in DMEM media without phenol red at 37°C in dark for 30 min. The photographs were then taken by inverted fluorescent phase contrast microscope and the mitochondrial depolarization patterns of cells for cells quantification were examined by using imaging software NIS-Elements F 4.00.00.

### DNA extraction and fragmentation assay

Assay was done by the method of [23] with minor modifications. At the end of treatment period, cells were harvested and washed twice with cold PBS. The cell pellets were lyzed in 500 µl DNA lysis buffer (20 mM EDTA, 10 mM Tris-HCl pH 8.0, 0.2% Triton X-100 and 100 µg/ml Proteinase K) for 1.5 h at 37°C. The samples were centrifuged at 6000×g for 5 min to collect the supernatant containing DNA. The supernatant was added with equal volume of isopropanol and 25 µl 4M NaCl to make 100 mM final concentration and incubated overnight at −20°C. Sample was then centrifuged at 6000×g for 25 min at room temperature. The pellet was dissolved in 50 µl ddH_2_O and 2 µl RNase A (10 mg/ml) and reincubated at 37°C for 1 h. The extracted DNA was subjected to electrophoresis on 1.5% agarose gel containing ethidium bromide. The DNA bands were read under ultraviolet illumination gel-doc system (QUANTUM-ST4-1326.WL/26MX XPRESS, France).

### Analysis of cellular DNA contents by flow cytometry

Cell cycle phase distribution with cellular DNA contents were carried out using flow cytometry. A431 cells were seeded into 6-well plate at density 1×10^6^ cells/ml and treated with 100 µM, 300 µM and 500 µM of naringenin for 24 h in 5% CO_2_ incubator and at 37°C temperature [24]. After 24 h incubation, the cultured cells were harvested, washed with cold PBS, fixed in 70% ethanol and treated with RNase A (10 mg/ml). Fixed cells were stained with propidium iodide (PI) dye followed by incubation for 30 min at room temperature in dark. The PI fluorescence of individual nuclei was measured using flow cytometer (BD FACS Calibur, Becton Dickinson, USA). Data were analyzed with the Cell Quest Pro V 3.2.1 software (Becton Dickinson, USA).

### Analysis of Caspase-3 activity

The caspase-3 activity was assayed using Caspase-3 Colorimetric Assay Kit (Catalog No. K106, BioVision, USA). Approximately 3×10^6^ (treated and untreated) of cells were resuspended with chilled lysis buffer. The cell lysate was incubated on ice for 10 min prior to centrifugation (10,000×g for 1 min). The reaction buffer, with 10 mM DTT was added to the supernatant of cell lysate and incubated further for 30 min on ice. The cell lysate (50 µl) was aliquoted into wells of 96-well microplate and 50 µl of reaction buffer containing 10 mM DTT was then added to the lysate. About 5 µl of 4 mM DEVD-pNA substrate was added in each wells and incubated at 37°C for 2 h. Absorbance at 405 nm was then read in a microplate reader. The absorbance of treated cells was compared with untreated control to find the change in caspase-3 activity.

### Statistical analysis

Data were expressed as the mean ± SD from three independent experiments. One-way ANOVA and Dunnett’s multiple comparison test were performed using Graph Pad prism (Version 5.01) software for significance test, using a p value≤0.05.

## Results

### Effect of naringenin in normal HaCaT cells

**Figure 1:**
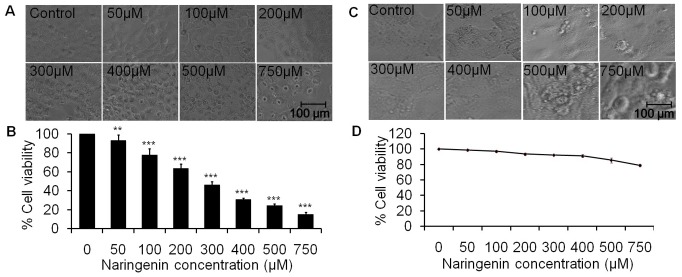
*In vitro* activity of naringenin against Human skin carcinoma A431 and normal HaCat cells. (A) Morphological view of live and dead cells of A431 cell line treated with 50 µM to 750 µM concentration of naringenin (B) The percent cell viability of A431 cells measured by a MTT assay at 24 h as described in the experimental section. (C) Morphological analysis of normal HaCat cell line treated with 50 µM to 750 µM concentration of naringenin. Photomicrographs were taken by inverted phase contrast microscope. (D) The percent cell viability of HaCat cell measured by a MTT assay at 24 h. Values are expressed as means ± SD of at least three independent experiments, **p<0.01 and ***p<0.001 as compared with their respective control.

To find out the experimental doses of naringenin, cytotoxic tests were performed on normal skin cells HaCaT using MTT assay and cell morphology assessment. Morphological study suggests that higher concentration 750 µM of naringenin changes cell morphology and induces cell death. However, doses 50 µM, 100 µM, 200 µM, 300 µM, 400 µM and 500 µM of naringenin did not affect significantly on cell morphology as revealed in Fig. 1C. Cell viability data showed that percent cell viability in normal cells was 91.97% and 85.40% at 400 µM and 500 µM concentrations of naringenin respectively i.e. cell death was statistically insignificant, however, significant cell death was observed at 750 µM of naringenin (Fig. 1D).

### Naringenin induces morphological changes and inhibits cell viability of A431 cells

The cell morphology was assessed using inverted phase contrast microscopy under 20× objective (Fig. 1A). Photograph clearly revealed that morphological shapes of the cells were drastically changed being round in shape in the treatment groups which is characterized by cellular shrinkage, detachment from surface and deformation of cell bodies at higher concentrations. A dose dependent cell morphological changes were observed in A431 cells at 24 h treatment of naringenin. The effect of different concentrations of naringenin on cell viability was shown in Fig. 1B. The percent cell viability data indicates that at 50 µM, 100 µM and 200 µM of naringenin concentrations, cell viability reduced to 93.01%, 77.92%, and 63.69% respectively as compared to control, which further dropped to 46.35%, 30.82%, 24.47% and 15.44% (p<0.001) at 300 µM, 400 µM, 500 µM and 750 µM, respectively. Results revealed that last three doses are more cytotoxic. We have selected three optimum doses (100 µM, 300 µM and 500 µM) for further studies.

### Naringenin causes nuclear changes with apoptosis within A431 cells

**Figure 2:**
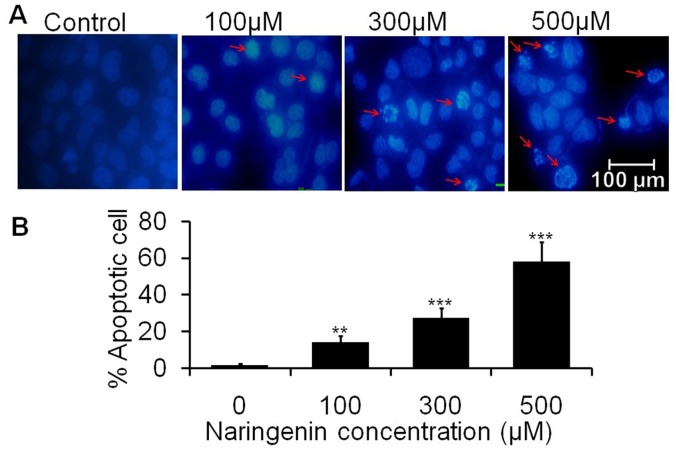
Chromatin condensation of A431 cells stained with DAPI after naringenin treatment. Cells were treated with 100 µM, 300 µM and 500 µM of naringenin. (A) Photomicrographs were taken by florescence phase contrast microscope that showed fragmented and condensed nuclei as indicated by arrow (B) Numerical data were expressed as % apoptotic cells respective to their control. Data is representative of three independent experiments and expressed as means ± SD, **p<0.01 and ***p<0.001 as compared with their respective control.

Nuclear apoptosis were observed by using florescent DAPI dye. Condensed and fragmented nuclei were regarded as apoptotic cells. As observed from photomicrograph, naringenin induces the nuclear condensation in a dose dependent manner as indicated by arrow (Fig. 2A). Maximum chromatin condensation was observed in the cells treated at 500 µM of naringenin. Furthermore, approximately 14.00% and 27.30% and 58.00% apoptotic cells were observed at 100 µM, 300 µM and 500 µM of naringenin, respectively (Fig. 2B).

### Naringenin induces intracellular ROS generation in A431 cells

**Figure 3:**
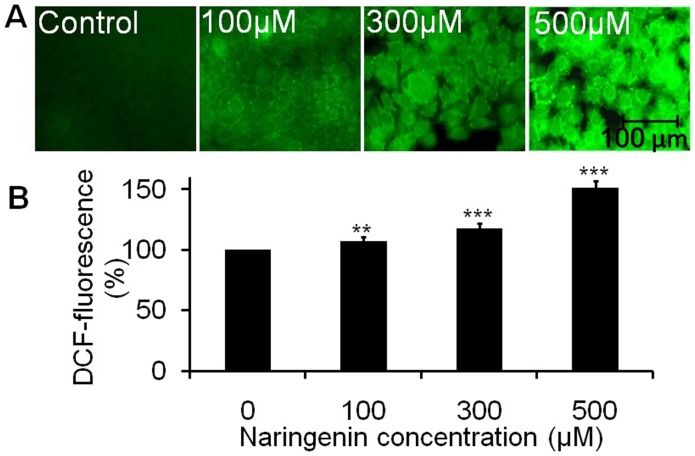
Photomicrographs showing intracellular ROS generation in A431 cells induced by naringenin and stained with DCFH-DA. (A) Photomicrographs showing intracellular ROS generation induced by 100 µM, 300 µM and 500 µM of naringenin after 12 h incubation. Photomicrographs were taken by florescence phase contrast microscope (B) Values are expressed as the percentage of fluorescence intensity relative to the control. Values are expressed as means ± SD of at least three independent experiments, **p<0.01 and **p<0.001 as compared with their respective control.

ROS generation was assessed at 12 h after naringenin treatment. Florescent micrograph of stained A431 cells (Fig. 3) depicts the effect of naringenin-induced intracellular ROS generation. The photomicrograph suggests that A431 cells treated with naringenin elevates ROS intensity in a dose dependent manner as compared to control (Fig. 3A). Maximum ROS generation was observed in the cells treated at 500 µM of naringenin. The quantitative measurement of ROS intensity showed that 100 µM of naringenin elevates significant ROS production, approximately 7.43% (**p<0.01) as compared to control. However, ROS production was sustained increasingly to approximately 17.46% and 51.52% (**p<0.001) at the concentrations 300 µM and 500 µM of naringenin, respectively (Fig. 3B). Increased ROS generation is involved in the induction of apoptosis through various pathways.

### Naringenin modulates mitochondrial membrane potential (ΔΨm) of A431 cells

**Figure 4:**
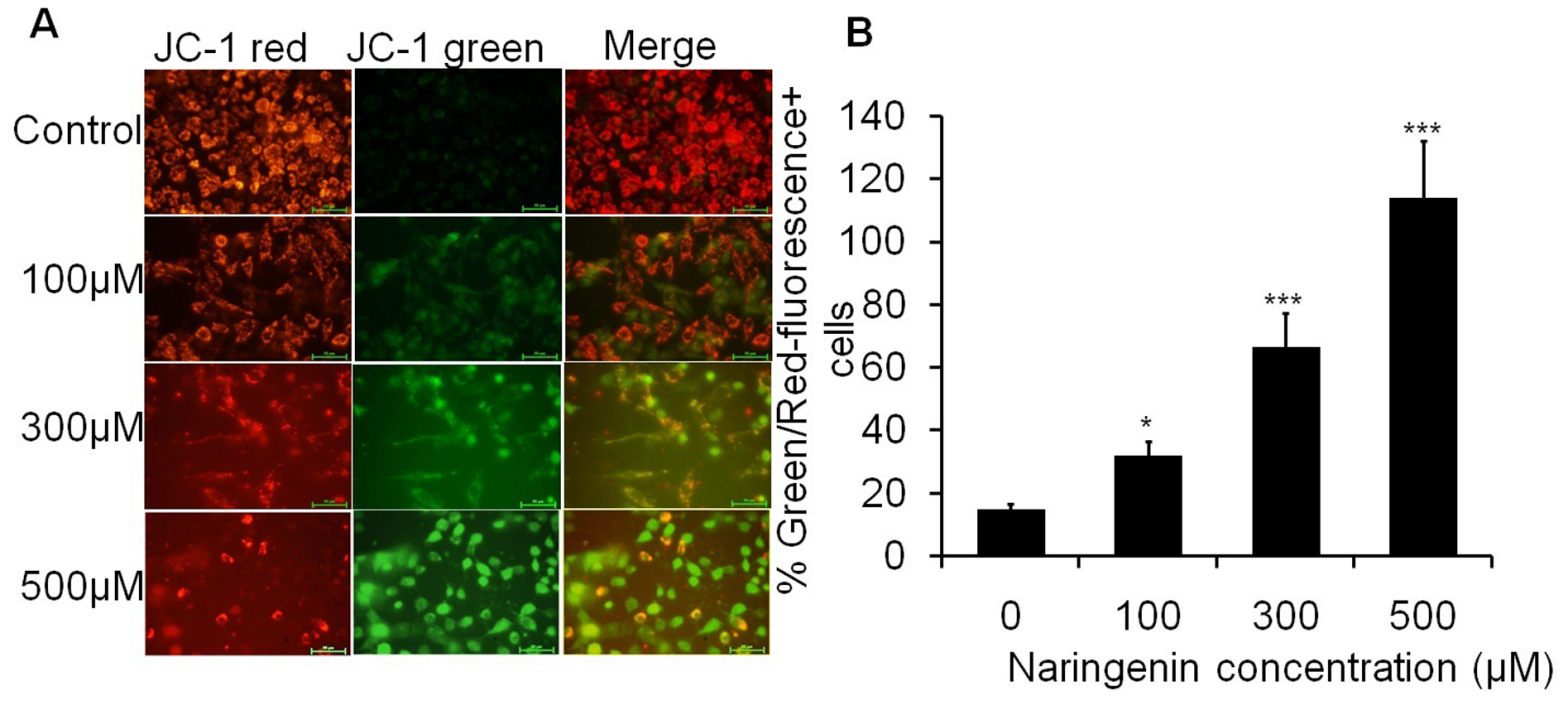
Fluorescence image of A431 cells stained with JC-1 after 24 h incubation with different concentrations of naringenin. (A) Photograph showing JC-1 red, JC-1 green and merge image. The JC-1 green fluorescence indicates a decrease in mitochondrial membrane potential, an early event in apoptosis. Increased concentrations of naringenin attenuated the loss of mitochondrial membrane potential. (B) Numerical data were expressed as % Green/Red fluorescence^+^ cells which were increased with increasing doses of naringenin. Data is representative of three independent experiments and expressed as means ± SD, *p<0.05, **p<0.01 and ***p<0.001 as compared with their respective control.

The increased and decreased florescent intensity of red and green florescent caused by JC-1 indicates the change in mitochondrial membrane potential (*ΔΨ*m). Result indicated that cells treated with 100 µM, 300 µM and 500 µM of naringenin induced a strong green fluorescence in a dose dependent manner (Fig. 4A). The merge images of JC-1 red and JC-1 green cell treated with 500 µM of naringenin showed that majority of cells express strong green fluorescence, indicating potent apoptotic activity of naringenin. As shown from quantitative data, the Green/Red-fluorescence^+^ cells ratio were found to be 31.68%, 66.25% and 113.80% at 100 µM, 300 µM and 500 µM of naringenin treatment, respectively (Fig. 4B).

### Naringenin induces DNA fragmentation of A431 cells

**Figure 5:**
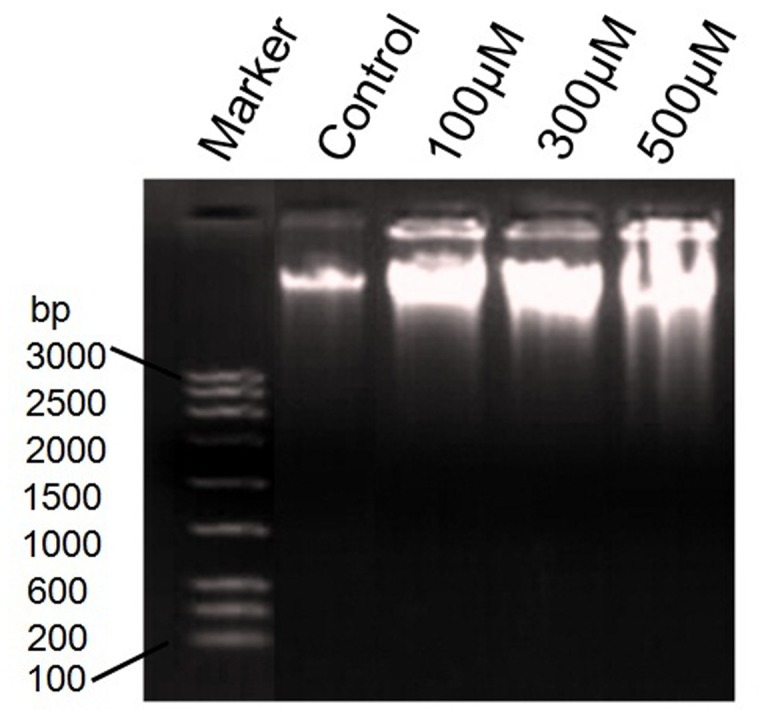
DNA fragmentation analysis. DNA fragmentation of A431cell treated with 100 µM, 200 µM and 300 µM of naringenin. 1^st^ well showing the 3 kb DNA marker. DNA laddering pattern showed that naringenin induces DNA fragmentation in a dose dependent manner.

Naringenin was tested to ascertain the DNA damage following exposure of various concentrations of compound to A431 cells. The results obtained from the DNA fragmentation assay (Fig. 5) showed the undamaged DNA in control cells whereas naringenin treated cells represent DNA fragmentation which was increased in a dose dependent manner. Small shearing was observed in the cells treated with 100 µM and 300 µM of naringenin, though maximum fragmentation was observed at 500 µM of naringenin exposure.

### Naringenin modulate cellular DNA content and induces G_0_/G_1_ phase arrest

**Figure 6:**
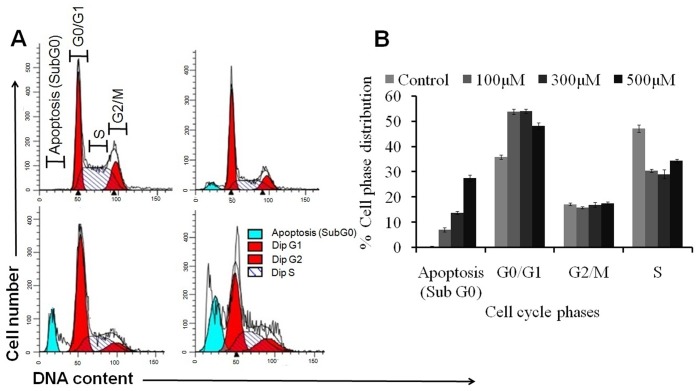
Effect of naringenin on different phases of cell cycle. A431 cells were treated with different concentrations of naringenin (100 µM−300 µM) for 24 h, stained with propidium iodide and measured by flow cytometry (A) Representative photomicrograph showing the apoptosis and phase distribution of cell population (B) Quantitative distribution of percentage of A431 cells in different phases of cell cycle treated with different concentrations of naringenin. Data is representative of three independent experiments.

Cell cycle analysis with cellular DNA content was performed by flow cytometry. Apoptotic cells were estimated by calculating the number of sub-diploid cells in the cell cycle histogram. When cells were exposed to 100 µM of naringenin, apoptotic cells reported were 6.24% as compared to control. However, at 300 µM and 500 µM of naringenin treatment, apoptotic cells were markedly increased to approximately 14.39% and 26.32% respectively. Analysis of cell cycle revealed that the cells in G_0_/G_1_ phase were observed to be 35.86%. However, 100 µM of naringenin arrest the hypodiploid cells approximately 53.75% in G_0_/G_1_ phase while 300 µM and 500 µM of naringenin arrest the cells 54.03% and 48.20% as compared to control (Fig. 6A and 6B).

### Naringenin induces caspase-3 activity in A431 treated cells

**Figure 7:**
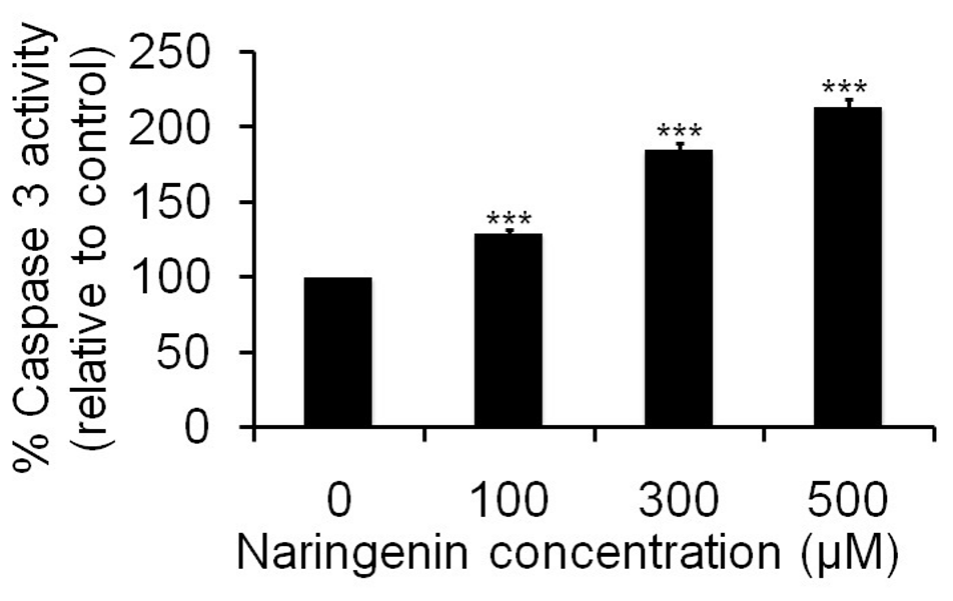
Activation of caspase 3 by naringenin against human skin carcinoma A431 cells. Cells were incubated with naringenion at 100 µM, 300 µM and 500 µM for 24 h. Values are expressed as means ± SD of at least three independent experiments, **p<0.01 and ***p<0.001 as compared with their respective control.

The activity of caspase (cysteine-dependent aspartate-specific proteinase), an important biochemical feature in apoptotic signaling, was further investigated to determine whether the apoptosis was induced by naringenin. Caspase-3 is the main downstream effecter caspase in apoptotic pathway and hence we compared its activity in treated and untreated control cells. The result showed that caspase-3 was significantly increased to 29.57% and 85.10% (***p<0.001) at concentrations 100 and 300 µM of naringenin. However, caspase-3 activity was dramatically increased to 114.02% (***p<0.001) at 500 µM naringenin concentration (Fig. 7).

## Discussion

Phyto-chemicals have been demonstrated significant potential as anticancer therapeutic agents due to their ability to inhibit tumor growth, angiogenesis, and metastasis without many side effects [25]. Much of these activities come from flavonoids, which are principal components of many such phyto-chemicals and demonstrate the capacity to inactivate carcinogens, inhibit angiogenesis, and halt cell proliferation and promote apoptosis [26]. Naringenin is a flavonoid abundant in citrus fruits, especially their peels and rinds, and has been suggested to exhibit antiproliferative effects against human tumor cells. As naringenin is generally present in foods bound to sugars as b-glycosides (i.e., naringenin), it was originally thought that absorption from the diet would be negligible. However, a number of studies have detected naringenin in human urine and plasma following oral doses of pure naringenin or the grapefruit juice [27], [28], [29], [30].

Here we have endeavored to work out the effects of the naringenin on epidermoid carcinoma A431 cell line. Cell viability analysis suggests that naringenin halts proliferation of cells (Fig. 1 B). Further morphological analysis of photomicrograph by phase contrast microscopy (Fig. 1A) showed that the morphological shapes of the cells were drastically changed being round in shape in the treatment groups which is characterized by cellular shrinkage and detached from surface, forming clusters. These results suggested that the treatment of naringenin significantly reduces the cell viability of cancer cell line in a dose dependent manner.

The accumulation of free radical mediated damage may be a possible mechanism of cancer development [31]. Excessive ROS production can lead to oxidation of macromolecules and play an important role in mtDNA mutations, ageing, and cell death [32]. Therefore, increased intracellular ROS production might be implicated in cellular damage of skin carcinoma. Our experiments for the qualitative and quantitative analysis of ROS generation suggest that naringenin induced ROS mediated apoptosis induction in A431 cells. As compared the cell viability assay to ROS generation assay inhibiting cell viability and induction of apoptosis, both mechanisms showed similar trends. Mechanistically, it is well understood that the ROS damage almost all classes of sub-cellular components, are produced in numerous patho-physiological states and have been recognized as participating in the development of multistage carcinogenesis [33]. Oxidative stress is associated with damage to a wide range of micro and macromolecular species including lipids, proteins, and nucleic acids, thereby, producing major interrelated derangement of cellular metabolism including peroxidation of lipids, formation of protein carbonyls, and single strand breaks in DNA [34]. In addition, the intrinsic increase of basal ROS in cancer cell are highly dependent on antioxidant enzymes systems *viz.* glutathione (GSH) and glutathione peroxidase (GPX) under the influence of anti-oxidant agents which perturb the anti-oxidant system and lead apoptosis [35]. Previous report has demonstrated the dietary agent could effectively inhibited B(a)P induced lung carcinogenesis by offering protection from protein damage and also by suppressing cell proliferation [36]. Fragmented and condensed nucleus in the cells is a hallmark of apoptotic induction [37]. Our result also supporting such statement where fragmented and condensed nuclei stained with DAPI dye in A431 cells suggested that naringenin caused cell death by an apoptotic process. The loss of mitochondrial membrane potential (*ΔΨ*m) is as an early event in apoptotic process as shown by earlier workers [38], [39]. Our data also support this finding as shown in Fig. 4A and 4B that naringenin depolarizes the membrane potential significantly in a dose dependent manner. The results indicate that the sub fraction of naringenin possesses promising apoptotic potential.

DNA fragmentation is generally considered to be the hallmark of apoptosis. Our DNA fragmentation data support earlier observation that naringenin induced the DNA fragmentation and subsequent cellular damage (Fig. 5). Indeed, the nuclear DNA of apoptotic cells shows a characteristic laddering pattern of oligonucleosomal fragments. Several studies have demonstrated a positive correlation between DNA fragmentation and apoptosis [22], [24].

The results support the findings that key changes in cell death can occur without DNA fragmentation and it alone should not be considered as a criterion for assessing apoptotic cell death [40]. To test the other parameters for apoptosis, we have performed cell cycle kinetic assay and observed that naringenin have antitumor actions, causing the cell cycle arrest, induction of apoptosis or a combination of these mechanisms, as earlier workers have also reported similar finding through different methods [41], [42]. In order to elucidate the type of death induced by naringenin, apoptotic characteristics including hypodiploidy (sub-G_1_ peak) of the cells were analyzed by flow cytometry using PI staining. G_1_ and G_2_ phases of the cell cycle are the major check points and have an important role in cell cycle progression [43]. Result of cell cycle study indicates that naringenin arrests the cells in G_0_/G_1_ phase of cell cycle and consequently lead to apoptosis (Fig. 6A and 6B). Caspase-3 is the primary effector caspase of the cell and plays an important role in the execution of apoptotic cell death by cleaving the cellular substrates [44], we then compared its activity in treated and untreated cells which favors that naringenin induced the caspase-3 activities and induction of caspase-3 leads to cell apoptosis (Fig. 7).

In conclusion, the present study provides a novel insight into the mechanism of action of naringenin-induced apoptosis in human skin carcinoma cells without/little affecting normal skin cells and shows a link between antiproliferative and apoptotic induction, and the cell death was due to the induction of ROS mediated mitochondrial membrane depolarization, nuclear condensation and DNA fragmentation. We also observed that naringenin induced cell cycle arrest at G_0_/G_1_ phase and caspase-3 activity. Our data confirm the potential of naringenin as an agent of chemotherapeutic and cytostatic activity in human skin carcinoma cancer and therefore, may be potentially valuable for application in drug developments. For further confirmation, molecular mechanism will be carried out to elucidate the molecular pathways.
